# Delayed Rectifier and A-Type Potassium Channels Associated with Kv 2.1 and Kv 4.3 Expression in Embryonic Rat Neural Progenitor Cells

**DOI:** 10.1371/journal.pone.0001604

**Published:** 2008-02-13

**Authors:** Dean O. Smith, Julie L. Rosenheimer, Ronald E. Kalil

**Affiliations:** 1 Stem Cell Research Laboratory, University of Hawaii at Manoa, Manoa, Hawaii, United States of America; 2 Department of Anatomy, University of Hawaii at Manoa, Manoa, Hawaii, United States of America; 3 Department of Ophthalmology and Visual Sciences, University of Wisconsin–Madison, Madison, Wisconsin, United States of America; Columbia University, United States of America

## Abstract

**Background:**

Because of the importance of voltage-activated K^+^ channels during embryonic development and in cell proliferation, we present here the first description of these channels in E15 rat embryonic neural progenitor cells derived from the subventricular zone (SVZ). Activation, inactivation, and single-channel conductance properties of recorded progenitor cells were compared with those obtained by others when these Kv gene products were expressed in oocytes.

**Methodology/Principal Findings:**

Neural progenitor cells derived from the subventricular zone of E15 embryonic rats were cultured under conditions that did not promote differentiation. Immunocytochemical and Western blot assays for nestin expression indicated that almost all of the cells available for recording expressed this intermediate filament protein, which is generally accepted as a marker for uncommitted embryonic neural progenitor cells. However, a very small numbers of the cells expressed GFAP, a marker for astrocytes, O4, a marker for immature oligodendrocytes, and βIII-tubulin, a marker for neurons. Using immunocytochemistry and Western blots, we detected consistently the expression of Kv2.1, and 4.3. In whole-cell mode, we recorded two outward currents, a delayed rectifier and an A-type current.

**Conclusions/Significance:**

We conclude that Kv2.1, and 4.3 are expressed in E15 SVZ neural progenitor cells, and we propose that they may be associated with the delayed-rectifier and the A-type currents, respectively, that we recorded. These results demonstrate the early expression of delayed rectifier and A-type K^+^ currents and channels in embryonic neural progenitor cells prior to the differentiation of these cells.

## Introduction

Embryonic neural progenitor cells (eNPC) from the developing brain have been isolated, cultured, and shown to differentiate into neurons, astrocytes and oligodendrocytes [1], [2]. By culturing these cells *in vitro* in defined media, the cellular and molecular aspects of neural differentiation can be analyzed under controlled conditions [3].

Neurons derived in culture from rat and human eNPC cells express voltage-gated ionic currents indicative of physiological function [4]. With respect to voltage-activated K^+^ currents, as many as three components have been recorded from eNPC [4], including non-inactivating delayed rectifier and faster 4-aminopyridine (4AP)-sensitive inactivating A types. In cells derived from very young rat embryos (E10.5), putative inwardly rectifying currents also were evoked [5]. Na^+^ currents have been recorded from many immature cells [6] but not from pluripotent mouse or human embryonic stem cells [7]. The Na^+^ currents recorded were mainly tetrodotoxin-sensitive, as in adult animals, but current density was generally low. Only rarely was the current density sufficient to generate an action potential [6]. Similarly, action potentials were not detected in immature neurons derived from progenitor cells in the embryonic (E20) rat cerebellum [8] or in cells from the adult rat subventricular zone [9].

Current density may depend on a cell's differentiation or proliferation state. For example, it has been shown in forebrain eNPC derived from newborn rats that Na^+^ and K^+^ current density increases under conditions that promote differentiation, such as substrate attachment, when compared with currents in presumably undifferentiated dissociated cells [10]. In contrast, when embryonic hippocampal progenitor cells are cultured under conditions that foster proliferation low levels of Na^+^ currents are observed [9].

Despite the wealth of information available about the properties of ligand- and voltage-gated channel properties during the normal development of the nervous system [11], surprisingly little is known about specific channel expression in cultured eNPC other than the basic current responses mentioned above. To our knowledge, there has been only a single cDNA microarray analysis of eNPC [5]. Most ion channel genes were not detected, but three Na^+^ and 13 K^+^ channel genes were resolved. Among these genes were those that encode delayed rectifier, inwardly rectifying, and Ca^2+^ sensitive K^+ ^channels. Unfortunately, no further attempts were made to relate those currents with expressed channel types. More recently, Kv 1.3 and 3.1 channels were identified in eNPC in a study that focused on the Kv 1 and Kv 3 groups of channels [4].

Here we have endeavored to define some of the relations between current properties and channel expression in eNPC derived from the embryonic rat (E15) subventricular zone (SVZ). We have focused on neuronal K^+^ channels in undifferentiated eNPC, because of their importance during embryonic development [11], [12] and in cell proliferation [4], [13]. Western blotting and immunocytochemical staining demonstrated the expression of Kv 2.1 and 4.3. In whole-cell recordings, we detected outward currents comprised of two components, a delayed rectifier and an A-type. Based on our evidence, we propose that Kv 2.1 and 4.3 may be associated with delayed-rectifier and A-type currents, respectively.

## Methods

### Cell Derivation and Culture

Neural progenitor cells were isolated from the SVZ of E15 embryos from timed-pregnant Sprague-Dawley rats. The rats were deeply anesthetized with pentobarbital sodium, and a midline incision was made to expose the uterine horns. The head of each embryo was removed, washed twice in chilled Hibernate E medium (BrainBits, Springfield, IL) supplemented with penicillin G (100 U/ml), streptomycin (100 µg/ml) and amphotericin B (2.5 µg/ml), and then placed in the same medium for subsequent dissection of the brain. The lateral and medial ganglionic eminences comprising the SVZ of each hemisphere were dissected under a surgical microscope and transferred to cold Hank's balanced salt solution (HBSS) with 1 mM EGTA and without Ca^2+^ or Mg^2+^. Dispase (2 U/ml) and trypsin (0.05%) were added, and the tissue then was triturated through a pipette before incubating at 37°C for 10 minutes.

The cells and debris were spun down, resuspended in HBSS with 10 mM MgCl and 10 units of DNase, and incubated at 37°C for 10 minutes with gentle agitation. Debris was removed by passing the disaggregated tissue through a 20 µm pore Nitex filter (Sefar, Ontario, CA). Next, the resulting single cell suspension was centrifuged, and the cells were resuspended in “complete” culture medium that consisted of 96% Neurobasal medium (Gibco) supplemented with 2% retinoic acid-free B27 (Gibco, Carlsbad, CA), 1% glutamine (GlutaMAX, Gibco), 1% penicillin/streptomycin (Gibco), 20 ng/ml epidermal growth factor (Peprotech, Rocky Hill, NJ), and 20 ng/ml basic fibroblast growth factor (FGF-2; Peprotech ).

The cells then were expanded in complete medium. After this expansion, an aliquot of cells was plated onto both untreated tissue-culture dishes (Falcon) and glass-bottom chamber slides (Lab Tek II, Nalgene Nunc International, Rochester, NY) for electrophysiological recording and immunocytochemical staining, respectively. Thus, both sets of experiments were performed on cells from the same culture. In initial trial experiments, some dishes and slides were coated with 0.1 to 1 mg/ml of either poly-L-lysine (Sigma, St. Louis, MO) or poly-L-ornithine (Sigma). The poly-L-ornithine (1 mg/ml) appeared to induce the fastest growth [14], although we did not quantify this. *Post hoc* analyses of these preliminary studies failed to detect any significant differences in results that might have depended on these substrata. Thus, the results reported in this study were obtained from cells cultured in these treated and untreated dishes and glass-bottom slides. The cells were plated at a density of 50,000/cm^2 ^and incubated in 5% CO_2_ at 37°C. They proliferated *in vitro*, and if they approached confluence, they were detached using 0.05% trypsin-EDTA (Gibco), pelleted (1500 r.p.m. for 5 minutes), resuspended, and re-plated. When reporting days *in vitro*, we refer to time in culture relative to the derivation date.

### Immunostaining

All of the antibodies used in this study were obtained from Millipore (Temecula, CA). They were directed against nestin (MAB353), βIII-tubulin (MAB1637), GFAP (MAB3402), NeuN (MAB377), Kv1.1 (AB5174), Kv1.2 (AB5176), Kv2.1 (AB5186), Kv3.1b (AB5188), Kv3.4 (AB5192), and Kv4.3 (AB5194).

#### Immunocytochemistry

For immunocytochemical staining, cells attached to coverslips were first rinsed twice in 0.1 M phosphate-buffered saline (PBS), fixed in 4% paraformaldehyde in 0.1 M PBS for 10 minutes at 4°C, and rinsed again in 0.1 PBS. The cells then were incubated at room temperature for 10 min in 0.2% Triton X-100, and, after rinsing in 0.1 M PBS, they were subsequently incubated in 5% goat serum for 30 min. Primary antibodies were applied overnight at 4°C. Following three rinses in 0.1 M PBS containing 0.05%TWEEN, the cells were incubated with a highly cross-adsorbed, fluorophore-conjugated secondary antibody (AlexaFluor 488 or 594, Molecular Probes, Carlsbad, CA) for two hours at room temperature. This procedure was repeated a when second primary antibody was applied. Controls for non-specific antibody binding utilized primary antibodies that had been immuno-adsorbed to antigen provided by the antibody vendor (Millipore). Coverslips were mounted on slides with Vectashield (Vector Laboratories, Burlingame, CA) containing DAPI to stain cell nuclei. The cells were viewed using a fluorescence microscope (Olympus BX51) equipped with a monochrome camera (Hamamatsu ORCA EG-AR). Colorization and manual counting of cell numbers were performed using Metamorph (Universal Imaging, Sunnyvale, CA). In some merged images, brightness and/or contrast of one of the two individual images was adjusted to match intensities.

The relative proportions of cells expressing a particular cell-specific marker were estimated by dividing the number of cells that stained positively for the cell-specific marker by the total number of DAPI-stained nuclei. Data for cells expressing cell-specific markers were obtained from at least 200 cells sampled at three different sites from cultures maintained between 16 and 51 days *in vitro*.

#### Western Blotting

Cells in culture were rinsed with 0.1M PBS, trypsinized, pelleted (180g for 5 min) and washed 2x in ice-cold PBS. To prevent proteolysis, all subsequent incubations and spins for lysate preparation were carried out at 4°C or on ice, unless indicated otherwise. Cells (10^7^ cells/ml) were incubated in lysis buffer (150-mM NaCl, 80-mM NaF, 20-mM iodoacetamide, 100-mM HEPES, 0.5% Triton X-100, 250-µM PMSF, 1-mM Na-orthovanadate, 1.3-mM AEBSF, 1-µM aprotinin, 30-µM leupeptin, 500-µM bestatin, 20-µM pepstatin and 20-µM E-64) for 1 hr, followed by centrifugation (13,000g for 5 min) to remove nuclei and cellular debris. Lysate protein concentration was adjusted to 2-5 mg/ml following a colorimetric protein determination assay (*DC* Protein Assay, Bio-Rad). The lysate then was precipitated in acetone (lysate:acetone at 1:1.4; −20°C for at least 1 hr followed by centrifugation at 13,000g for 5 min), and the pellet was resuspended in Laemmli sample loading buffer. Samples (50 µg protein in 30-µl Laemmli buffer) were heated at 95°C for 5 min, resolved by 10% SDS-PAGE (50 µg/lane), and electro-transferred to PVDF. Membranes were blocked at room temperature for 1 hr in 0.1-M PBS with 3% goat serum (0.25 ml/cm^2^ membrane) and washed 2×5 min in PBS with 0.1% TWEEN 20 (PBST; 0.25 ml/cm^2^ membrane). They were then incubated overnight at 4°C with affinity-purified primary antibodies (Millipore) diluted 1:250 in PBST (0.1 ml/cm^2^ membrane) followed by 5×5-min washes with PBST and incubated at room temperature for 30 min with HRP-conjugated affinity-purified secondary antibodies (Bio-Rad, Hercules, CA) diluted 1:10000 in PBST (0.1 ml/cm^2^ membrane). For amplification and colorimetric detection of bands, the Bio-Rad Amplified Opti-4CN Detection Kit was used. Rat brain microsomal proteins (Upstate, Charlottesville, VA) were heated at 37°C for 30 min and resolved by SDS/PAGE (15 µg /lane) in adjacent lanes for positive controls. For negative controls, primary antibodies were preincubated at room temperature for 1 hr with the appropriate antigen supplied with the primary antibody prior to incubation with the membrane.

### Electrophysiology

**Figure 1:**
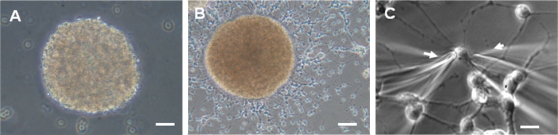
Neural progenitor cells. After forming a neurosphere, embryonic neural progenitor cells spread out into a monolayer. A. Neurosphere consisting of SVZ cells isolated at E15 that have aggregated in suspension after 2 days in culture. Scale bar: 100 µm. B. Neurosphere of SVZ cells derived at E15 that has attached to the floor of the culture flask after 4 days in culture. Note cells migrating away from the neurosphere. Scale bar: 100 µm. C. Cells at the periphery of neurospheres were chosen for electrophysiological recording. Most of the recorded cells extended processes. Arrows indicate the location of recording (left) and puffer (right) pipettes. Scale bar: 20 µm.

Recordings from undifferentiated eNPC were obtained at room temperature (23°C) at intervals that ranged form 20 to 50 days in culture. The cells were bathed in an extracellular solution containing (in mM) NaCl (130), KCl (4), CaCl_2_ (2), MgCl_2_ (1), HEPES (10), and glucose (10); the pH was adjusted to 7.35 with NaOH, and the osmolality was corrected to 295 using sucrose. Pipettes were drawn (DMZ Instruments) from 1.5-mm thin-walled filament glass capillaries (Harvard GC150TF). After fire-polishing (Narashige Instruments) the pipettes, the tips had a resistance of about 5 to 7 MΩ. The pipettes were filled with a solution containing (in mM) K gluconate (110), KCl (20), MgATP (2), Na-phosphocreatine (10), EGTA (1), GTP-tris (0.3), and HEPES (20). The pH then was adjusted to 7.25 with KOH, and the osmolality was corrected to 310 using sucrose. In some experiments, the pipettes were filled with a Cs solution containing CsCl (135), MgATP (4), EGTA (10) and HEPES (10); pH was adjusted to 7.25 with CsOH, and the osmolality was corrected to 310 using sucrose. Currents were recorded using a HEKA EPC10 patch-clamp amplifier in whole-cell mode (Figure 1 C). Record acquisition was controlled by PULSE software (HEKA Instruments), which corrected on-line for liquid-junction potential, leak currents (p/6 protocol), fast and slow capacitance, and series resistance (≥80%). Data were exported to IGOR (Wavemetrics, Inc.), Origin (OriginLab Corporation), or PULSE Tools (HEKA Instruments) software packages for subsequent analyses and curve fitting. Results are expressed as means±SEM. Cells were included in the analysis only if there was a consistent gigaseal and series resistance (<10 MΩ) throughout the experiment.

## Results

### Cytological Characteristics of Recorded eNPC

**Figure 2:**
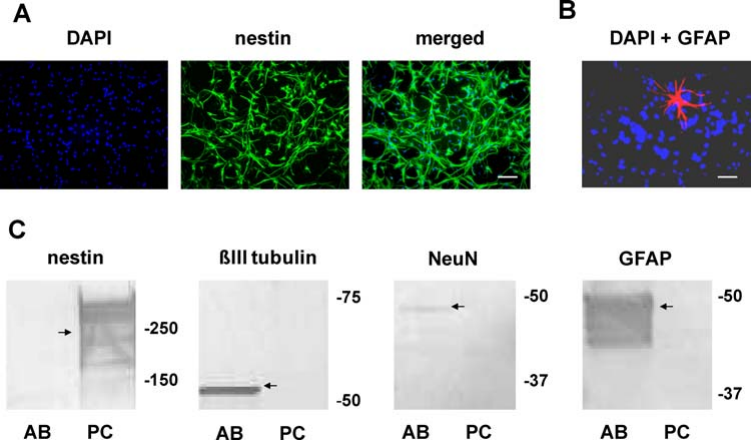
Undifferentiated cell phenotype. Embryonic rat neural progenitor cells were cultured in the presence of EGF and FGF-2. A. Immunocytochemical assays for nestin indicated that nearly all (>99%) cells expressed this intermediate filament protein associated with undifferentiated neural progenitor cells. Scale bar: 25 µm. B. After various intervals in culture small fractions of the cells, typically 0.5% or less, expressed GFAP, a marker for astrocytes, βIII tubulin (not shown), a marker for neurons, and O4 (not shown), a marker for oligodendrocytes. Scale bar: 50 µm. C. Western blots were prepared using control tissue obtained from an adult rat-brain microsomal preparation (AB) and total lysate from SVZ progenitor cells (PC). They indicated nestin expression in the progenitor but not the adult cells and, conversely, βIII tubulin and NeuN (a neuronal marker) in the adult but not the progenitor cells. The astrocytic marker GFAP was detected in the adult but only rarely the progenitor cells. The discrepancies between Western blotting and immunocytochemical staining with respect to resolving βIII tubulin, GFAP, and O4 expression in eNPC can be attributed to the greater sensitity of immunocytochemical staining in comparison to Western blotting.

Within 24–48 hours after seeding E15 eNPC derived from the SVZ in untreated tissue culture flasks, the cells aggregated to form neurospheres (Figure 1 A). During this time, some neurospheres attached to the surface of the flask, and cells migrated away from the spheres (Figure 1 B) to form a surrounding monolayer. We evaluated whether differentiation had occurred using immunocytochemical and Western-blot techniques (Figure 2). Immunocytochemical assays for nestin indicated that nearly all (>99%) cells expressed this intermediate filament protein, which is generally associated with undifferentiated neural progenitor cells (Figure 2 A). Very small fractions, typically 0.5% or less, of the cells expressed GFAP (Figure 2 B), a marker for astrocytes, βIII tubulin, a marker for neurons, and O4, a marker for immature oligodendrocytes. Immunocytochemical staining for βIII tubulin and O4 is not shown. In complementary experiments, Western blots were prepared using control tissue obtained from an adult rat-brain microsomal preparation or the total lysate from the SVZ eNPC (Figure 2 C). The blots indicated nestin expression in the eNPC but not the adult cells and, conversely, βIII tubulin, NeuN (a neuronal marker), and GFAP expression in the adult cells but not in the eNPC. Presumably the low-levels of GFAP and βIII tubulin expression that were detected in eNPC by immunocytochemistry were below the detection threshold for Western blotting.

### Voltage-gated K^+^ channel expression: immunostaining

At least 38 different voltage-gated K^+^ channel genes (the Kv families) have been identified [15]. Of these, 11 encode delayed rectifiers and 6 encode A-type channels [16]. We obtained antibodies and antigen for immunostaining controls to 6 of these channels, Kv 1.1, 1.2, 2.1, 3.1b, 3.4, and 4.3, that occur in the developing rodent brain [17] and tested for their expression using immunocytochemistry.

**Figure 3:**
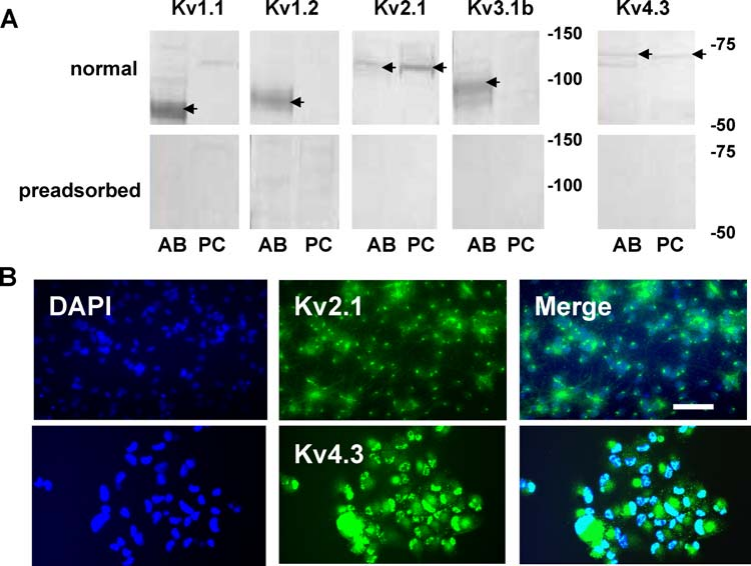
K^+^-channel sub-type expression. Western blots and immunocytochemical staining techniques were used to determine K_V_-channel expression. A. Western blots were prepared using control tissue obtained from an adult rat-brain microsomal preparation (AB) and total lysate from eNPC (PC). Results are shown from the normal assay (normal) and the assay following preasdsorption of the antibodies with antigen (preadsorbed). In the adult, positive staining was detected for Kv 1.1, 1.2, 2.1, 3.1b, and 4.3. However, in eNPC only Kv 2.1 and Kv 4.3 expression was detected consistently. B. Kv2.1 and Kv4.3 were also detected with immunocytochemistry. Images were obtained following staining with fluorescently labeled antibodies. The cell nuclei (DAPI), the sub-types, and the merged images of both are shown. The calibration bar is 100 µm for K_V_ 2.1 and 50 µm for Kv 4.3,

Western blots of adult rat brain microsomal proteins indicated positive staining for Kv 1.1, 1.2, 2.1, 3.1b, and 4.3 (Figure 3). However, in eNPC only Kv 2.1 and Kv 4.3 expression was detected consistently. Kv 3.4 was not detected consistently in either adult or eNPC. In these experiments, pre-adsorption of antibodies with their specific control antigens eliminated positive immunostaining. Immunocytochemical imaging of eNPC further demonstrated Kv 2.1 and 4.3 expression.

### Voltage-gated K^+^ channel expression: electrophysiology

Other K^+^ expression studies have indicated that Kv 2.1 encodes delayed-rectifier channels and that Kv 4.3 encodes A-type channels [15]. In view of this, we next tested for delayed-rectifier and A-type voltage-gated K^+^ currents using electrophysiological methods. The electrical properties of the K^+^ channels that were recorded in eNPC are compared with those measured in expressed Kv 2.1 and Kv 4.3 in Table 1.

**Table 1:** Summary of K^+^ channel electrical properties.

Channel type	Conductance		Activation			Inactivation	
	pS	V_a_ (mV)	k_a_ (mV)	τ(ms)	V_h_ (mV)	k_h_ (mV)	τ(ms)
Delayed rectifier	30.11±7.95	9.00±0.81	13.05±0.81	1.69±0.04	15.06±2.50	3.65±1.45	38.42±10.96
A-type	4.68±1.21	10.51±1.17	14.35±1.09	1.03±0.13	−34.75±1.33	8.87±1.80	15.97±0.92
	n = 6	n = 24	n = 23	n = 23	n = 6	n = 6	n = 12
Kv2.1	8.1±0.3^a^	11.39±3.80^b^	12.05±1.36^b^	13.3±0.3^c^	−25.2±0.8^b^	−5.8±0.5^b^	>500 c
Kv4.3	5 d	−27.1±1.4^ e^	6.5±1.2^ e^	1.7±0.2^ e^	−26.9±1.5^ e^	5.9±0.3^ e^	27.6±2.0 ,142±15^e ^(two components)

The means±standard errors are presented.

a [36]; ^b^ [35]; ^c^ [20];^ d^ [40];^ e^ [21]

#### Resting membrane properties

Immediately after attaining a stable whole-cell recording, the resting membrane potential was recorded in current-clamp mode. The values ranged from −78 to −20 mV (mean value, −46±2 mV, n = 51). Resting membrane capacitance, estimated by capacitance-canceling compensation in voltage-clamp mode, ranged between 5 and 15 pF (mean value, 9.1±0.7 pF, n = 36). Assuming 1 µF/cm^2 ^of membrane surface area, these values correspond to spherical cells with an average diameter of 18 µm, which is consistent with cell soma sizes observed under the microscope (Figure 1 C). None of these values varied with time *in vitro.*

#### Voltage-gated K^+^ currents

Voltage-gated currents were recorded from 42 cells. At membrane potentials ranging from −100 mV to +40 mV, inward currents were never detected. We concluded that Na^+^ currents were not present in undifferentiated eNPC.

**Figure 4:**
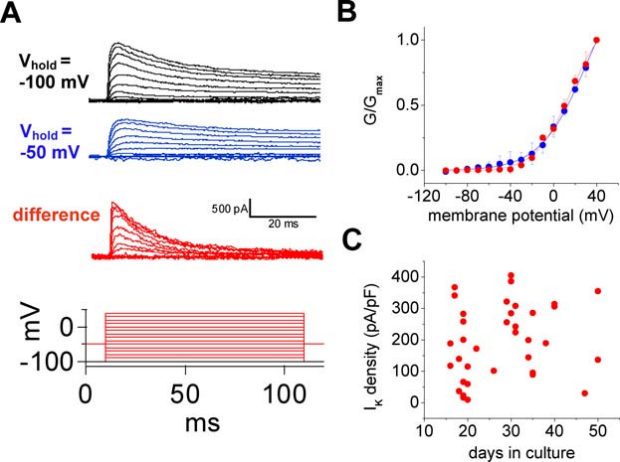
Voltage-gated outward currents. In whole-cell recordings, outward voltage-gated currents were detected. A. Currents were evoked in response to 10-mV membrane potential steps from −100 to +40 mV (bottom); holding membrane potentials were −100 (black) and −50 mV (blue). The differences between these currents recorded from the two holding potentials were calculated for each membrane-potential step (red). Two current types were identified: a fast-inactivating A-type, revealed by the subtraction protocol (red), and a more slowly inactivating delayed rectifier, obtained from the −50 mV holding potential (blue). B. The current-voltage curves for the two current components (red and blue) were plotted to illustrate activation properties. The conductance-voltage relationships were fit by Boltzmann equations (n = 23) to estimate activation parameters. C. Peak current density did not vary significantly as a function of the number of days in culture.

However, outward currents were observed in all cells (Figure 4). The peak amplitudes ranged from 0.1 to 4.8 nA (mean value = 2.0±0.2 nA) and the average current density, measured at peak amplitude, was 202±18 pA/pF. These values did not vary significantly with the number of days in culture (Figure 4 C). However, recorded currents were greatly reduced when CsCl replaced K-gluconate in the recording pipette solution, indicating that they are most probably K^+ ^currents. In contrast, neither α-dendrotoxin (≤80 nM) nor dendrotoxin-K (≤30 nM) affected the currents that were recorded.

**Figure 5:**
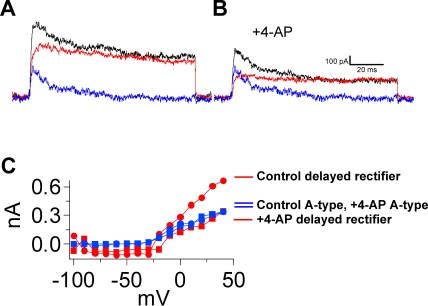
4-aminopyridine-sensitive voltage-gated potassium currents. The two current components responded differently to 4-aminopyridine (4-AP). A. Voltage-gated currents were evoked from a −100 (black) and a −50 (red) -mV holding membrane potential; the A-type current (blue) was derived by taking the difference between these two currents (black and red). B. The same currents were recorded 4 min after the addition of 10 µM 4-AP to the bath. The total (black) and the delayed rectifier (red) currents were reduced, but the A-type (blue) was unaffected. Thus, at the concentration of 4-AP that was used, only the delayed rectifier current is 4-AP sensitive. C. These differential sensitivities of the delayed rectifier (red) and A-type (blue) are further illustrated in the current-voltage relationships.

To test for multiple components, currents evoked by stepwise depolarization from −50 mV holding potential were subtracted from those evoked from −100 mV holding potential [18]. Two current types were identified: a fast-inactivating A-type, revealed by the subtraction protocol, and a more slowly inactivating delayed rectifier, obtained from the −50 mV holding potential (Figure 4 A). Interestingly, 10 µM 4-aminopyridine (4-AP) blocked the delayed-rectifier but not the A-type currents (Figure 5). At +50 mV, the delayed rectifier currents were reduced by 52±7% (n = 5, 0.05 level); changes in the A-type currents were not significant statistically (0.05 level). At concentrations >5 mM, both components were totally blocked.

#### Activation

Both current components had activation thresholds ranging from −40 to −30 mV (Figure 4). Activation time constants were estimated for each type by fitting an exponential curve to the rising phase; the two values, 1.69±0.04 and 1.03±0.13 ms for the delayed rectifier and the A-type, respectively, differ significantly (0.01 level). The delayed rectifier activation is considerably faster than in Kv 2.1 channels expressed in oocytes [19], but the A-type time constant is close to the value measured in expressed Kv 4.3 [20].

Activation voltage-dependence was determined from whole-cell conductance versus membrane potential measurements (V_m_). The whole-cell conductances, G, were estimated from current-voltage relationships using the formula G = I/(V_m_−V_reversal_). The reversal potential, V_reversal_, was assumed to equal the K^+^ equilibrium potential (−88.7 mV), which was calculated using the Nernst equation. The resulting curves were fit with Boltzmann functions (Figure 4 B), and the estimated values for the half-activation voltage (V_a_) and the slope factor (k_a_) are presented in Table 1. Neither of these two parameters differed significantly between the two current components (P>0.05 level). With one exception, they were also similar to those recorded in Kv 2.1 and Kv 4.3 when expressed in oocytes. The exception: A-type V_a _was considerably more positive than observed in Kv 4.3 when expressed in a mammalian cell line [21].

#### Inactivation

Inactivation kinetics were estimated by fitting single exponential curves to the decay phase of current responses following step depolarizations from the holding potential to +40 mV. The time constants were 38.42±10.96 ms and 15.97±0.92 ms for the delayed rectifier and the A-type currents, respectively (Table 1). These values differed significantly (one-tailed t-test, P<0.05 level).

**Figure 6:**
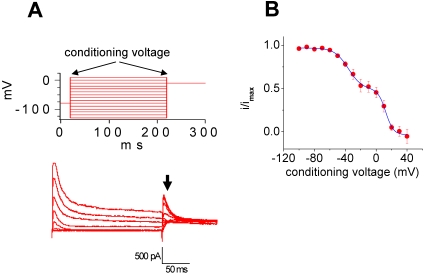
K^+^-current inactivation. The two current components had different inactivation properties. A. Inactivation properties were determined by applying 200-ms conditioning, inactivating membrane voltage steps from the resting level, −80 mV, to values ranging from −130 to +10 mV in 10-mV steps. The membrane potential was then set to 0mV. The peak amplitudes of currents evoked at this 0-mV test level were measured (arrow in lower panel) and normalized relative to their maximum value. B. The relative peak current amplitudes were plotted as a function of the conditioning voltage potential, and a double Boltzmann equation was fit to the data. The A-type inactivation parameters were comparable to those of Kv 4.3 when expressed in oocytes. However, the delayed rectifier and Kv 2.1 when expressed in oocytes differed in both inactivation kinetics and inactivation voltage dependence.

Voltage-dependent inactivation properties were determined by applying conditioning, inactivating membrane voltage steps from a −80 mV holding potential to levels ranging from −130 to +10 mV in 10-mV steps (Figure 6 A). The membrane potential was then set to 0 mV, thus activating any non-inactivated channels. The peak amplitudes of currents evoked at this 0-mV test level were then measured, normalized relative to their maximum value, and plotted versus the conditioning-voltage amplitude (Figure 6 B). The half-activation voltage (V_h_) and the slope factor (k_h_) were estimated by fitting a double-Boltzmann curve was fit to these data (Table 1). Both estimates differ significantly (0.05 level).

In general, the A-type inactivation parameters were comparable to those of expressed Kv 4.3. However, the delayed rectifier and expressed Kv 2.1 differed in both inactivation kinetics and inactivation voltage dependence.

#### Single-channel conductance

**Figure 7:**
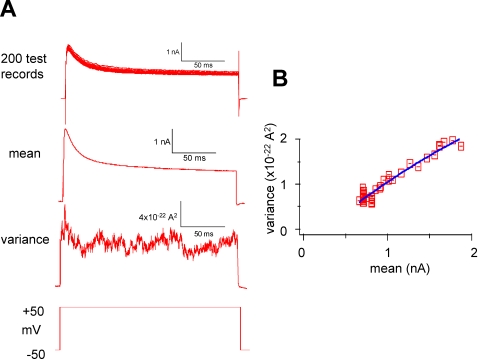
Single-channel conductance. Non-stationary noise analysis techniques were used to estimate single-channel conductance. A. Currents in response to stepping the membrane potential from −50 mV to +50 mV at least 200 times were recorded (upper trace). The mean and variance of these currents were then derived (middle traces). B. In a plot of the variance versus the mean (right), the data were fit with a second-order polynomial (blue curve) to estimate the single-channel current, *i*, and conductance as described in the Methods. The delayed-rectifier channel conductance is considerably larger than in expressed Kv 2.1. The 4.7 pS value is similar to the value in expressed Kv 4.3.

The conductance of single K^+^ channels was estimated by non-stationary noise analysis [22]. At least 200 whole-cell currents in response to 200-ms step depolarizations from the holding potential (−50 or −100 mV) to +50 mV were recorded. The mean (I) and variance (σ^2^) of these evoked currents were calculated, binned, and plotted against each other (Figure 7). These data are related by the parabolic relationship σ^2^−σ^2^_baseline_ = *i*. I^2^/N, where *i* and N represent the single-channel current and the total number of channels, respectively. Using PulseTools fitting routines (HEKA Instruments), *i* was estimated, and the single-channel conductance γ was calculated using the formula γ = *i*/(V_m_−V_reversal_).

The conductance values obtained from recordings at holding potentials of −50 and −100 mV were 30.1±8.0 and 4.7±1.2 pS, respectively. They differ significantly (P<0.05 level). The larger conductance, 30.1 pS, estimates the delayed-rectifier channel conductance. This is considerably larger than in Kv 2.1 when expressed in oocytes. The 4.7 pS value was derived from recordings with a −100-mV holding potential and, therefore, is a composite of both the delayed rectifier and the A-type channels. We conclude conservatively that the A-type single channel conductance is ≤4.7 pS, which is similar to the value in Kv 4.3 when it is expressed in oocytes.

#### Transformed Cells

**Figure 8:**
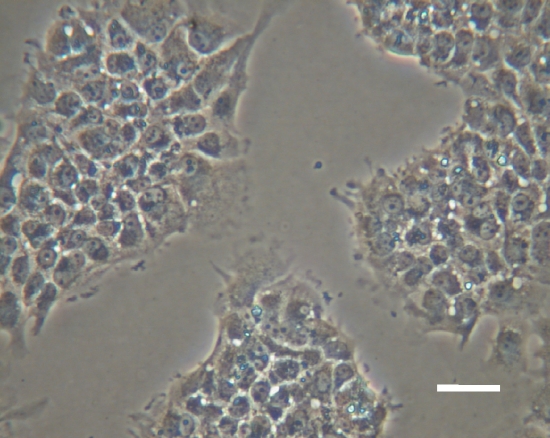
Transformed cells. The cells in this image were derived from the SVZ of an E15 rat embryo. The cells had been cultured continuously for more than two years and were still proliferating at the time of fixation. However, the cells had transformed at around 8 weeks in culture, and no longer resembled neural cells cytologically. Scale bar: 50 µm.

We attempted to maintain cells in complete media for long durations, but after about 56 days they often demonstrated dramatic cytological changes. In these cases, the cells became irregularly shaped and began to divide very rapidly (Figure 8). Karyotyping indicated that these cytological changes were associated with aneuploidy. Although the transformed cells had resting membrane potentials of about −50 to −60 mV, they did not display any voltage-gated currents.

## Discussion

In this study, we have presented a comprehensive description of voltage-activated K^+^ channels in E15 rat embryonic neural progenitor cells derived from the SVZ. We have demonstrated the expression of two different voltage-gated K^+^ currents, the delayed rectifier and the A-type. Moreover, we have identified two K^+^ channels that may be associated with these currents, namely Kv 2.1 and 4.3.

It has long been known that voltage-gated K^+^ currents occur ubiquitously in the developing nervous system [23]. They also appear in embryonic stem cells prior to [5] and following induction of neural differentiation *in vitro* [24], [25] and in different phenotypes as they emerge during differentiation at later developmental stages [8], [26]. Moreover, multiple K^+^ currents have been reported in rodent [4], [5], [27], [28] and human [29] eNPC. However, unlike most of these previous studies, we have focused our attention on eNPC that did not yet express markers of terminally differentiated cells. Since cells require voltage-gated K^+ ^channels to proliferate [4], [13], [30], [31] it is not unreasonable to speculate that supporting proliferation is the principal function of the K^+^ currents that we have observed in undifferentiated eNPC. The other major role for K^+^ currents, namely the regulation of neuronal excitability, would not be involved in these cells as they had not made a fate choice at the time of recording. Moreover, they had not yet developed Na^+^ currents capable of generating action potentials.

The rigor of these conclusions depends on several technical considerations: voltage-clamp control, antibody specificity, and the accurate identification of recorded cells. Despite careful efforts to establish full control of membrane voltage while in whole-cell recording mode, for example, great care was taken to correct for liquid-junction potential, leak currents, fast and slow capacitance, series resistance, etc., we cannot exclude the possibility of incomplete clamp in regions remote from the electrode tip. Nearly all cells that were recorded had long processes that resembled axons but were too thin to record from directly. Any voltage-dependent activity in these regions may not have been under complete voltage control and, in an unlikely instance, may have gone undetected.

The specificities and immunogens of each of the polyclonal anti-Kv antibodies that were used in this study for both immunocytochemistry and Western blotting have been well characterized by the vendor (Millipore). Moreover, the immunostaining for the Kv channels presented in Figure 3 has been reviewed by the vendor and the accuracy of the results confirmed (Lyndon M. Foster, Ph.D., Senior Manager Technical Information Services, Millipore Bioscience Division, personal communication). We further validated the specificity of the Kv antibodies that were used by preadsorbing each against its antigen, which eliminated subsequent binding by the antibody in Western blots and in immunocytochemical staining. While we cannot rule out completely non-specific binding of one or more of the antibodies used, the likelihood that such binding has confounded our results is negligible.

Our conclusions also depend upon the accurate identification of the cells in which K^+ ^currents were recorded. Ideally, every recorded cell would have been characterized according to type using immunocytochemical or other labeling methods. Despite previous success in this regard with primary cultured neurons [32], we were unable technically to label a sufficient number of single progenitor cells after whole-cell recordings in order to characterize them according to cell type. Nevertheless, immunocytochemical staining of attached cells indicated that nearly all of the cells expressed nestin but not βIII tubulin, GFAP, or O4. The few GFAP-positive cells, which were less than 1% of the total number of cells available for recording, typically were located near the middle of a large cell cluster (Figure 2 B). We never attempted to patch-clamp these cells because the overlapping cells in the cluster precluded visualization of the electrode tip. Furthermore, all of the individual cells from which recordings were made were located near the periphery of the cluster (as shown in Figure 1 C), and most appeared to be nearly identical cytologically under phase-contrast optics and immunocytochemically. There is always a chance that we recorded from a differentiated cell, but the immunocytochemical results argue that this is a not a significant possibility. Therefore, we are confident that the vast majority recordings that were made were obtained from nestin-positive, undifferentiated eNPC.

In a study comparable to the present one of Kv channel expression, Liebau et al. [4] identified three K^+^ currents, including a delayed rectifier and an A-type, and associated them with Kv 1.3, and Kv 3.1 expression in eNPC derived from the midbrain of E14.5 rat embryos. Our analyses clearly showed two K^+ ^current components, but we cannot rule out a third. We did not investigate Kv 1.3 expression, so we cannot comment on that observation. However, we did test for Kv 3.1b expression with immunocytochemistry and Western blotting, and detected it in adult tissue but not in eNPC.

At least two plausible reasons can be cited for the discrepancy between our observations and those of Liebau et al. [4] with respect to the existence of Kv 3.1 expression in eNPC. First, the sites from which eNPC were derived in our study (SVZ) and that of Liebau et al. [4, midbrain] are different, and during normal development they generate neurons with different functional roles. Secondly, in possible contrast to Liebau et al. [4], we incubated our cells in medium supplemented with retinoic acid-free B27 to avoid biasing the cultured eNPC toward neuronal differentiation. Moreover, while Liebau et al. [4] added EGF to the culture medium, we added FGF-2, which is not interchangeable with EGF, to the medium to promote cell proliferation. This difference in culture conditions and, therefore, in possible differences in the differentiation state of recorded cells may help to explain why our results and those of Liebau et al. [4] differ.

Can we relate channel expression to the two current components that we have described? At least 38 different voltage-gated K^+^ channel genes (the Kv families) have been identified to date [15], and there may be others. Given the large number of known K^+^ channels, we did not attempt to explore all possible K^+ ^channel types. Instead we focused on six K^+ ^channel candidates that *a priori* seemed likely to be expressed in eNPC from a functional perspective [17].

Because Kv 2.1 (also denoted *drk1*) is expressed prominently in granule cells of the adult rat olfactory bulb [33], we reasoned that it might also be expressed in eNPC derived from the SVZ, as these cells are the source of granule cells for the olfactory bulb. Of the two channel types that we identified, only Kv 2.1 encodes a delayed rectifier channel [34], [35]. When expressed in *Xenopus* oocytes, Kv 2.1 has considerably slower activation (τ_activation_ = 13 to16 ms) kinetics [19], [20] and a somewhat smaller single-channel conductance (8 pS) [36] than our estimates (2 ms and 30 pS, respectively). However, Kv 2.1 activation and other electrophysiological properties vary considerably depending on the expression system [37] and the phosphorylation state of the channel [38]. Therefore, it is not unexpected that the results that we report for Kv2.1 do not match precisely those reported by others for Kv2.1 expressed in *Xenopus* oocytes. Moreover, other K^+^channel types that we have not tested, such as Kv 2.2, 5.1, 6.1, 9.1, and 9.2, have been shown to modulate the electrophysiological properties of Kv2.1 considerably [19]. Thus, we conclude conservatively that Kv 2.1 is associated with the delayed rectifier current that we have recorded, but we cannot exclude the contribution of other channel types.

The Kv 4.3 channel is associated with an A-type current and is highly expressed in the olfactory bulb and other brain regions [39]. Thus, it is not unreasonable to speculate that Kv 4.3 channels mediate the A-type currents that we observed. The single channel conductance that we measured in eNPC was identical, 5 pS, to the value observed in Kv 4.3 channels expressed in a mammalian cell line [40]. Furthermore, the kinetic and voltage-dependent properties of expressed Kv 4.3 [21], [40], [41] resembled those of the A-type currents in eNPC with but one exception; although the activation threshold (approximately −30 mV) that we determined matched the values observed in expressed channels, the voltage dependence (Va) differed. Kv4.3 electrophysiological properties are modulated by K-channel interacting proteins (KChiPs) [40] and by co-expression with other K-channel subtypes, such as Kv 4.2 [42]. It is therefore possible that these or other factors may modulate the A-type current in eNPC that we have attributed to Kv4.3.
